# Band Structure and Intersubband Transitions of Three-Layer Semiconductor Nanoplatelets

**DOI:** 10.3390/nano10050933

**Published:** 2020-05-12

**Authors:** Ilia A. Vovk, Vladimir V. Lobanov, Aleksandr P. Litvin, Mikhail Yu. Leonov, Anatoly V. Fedorov, Ivan D. Rukhlenko

**Affiliations:** 1 ITMO University, Center of Information Optical Technology, Saint Petersburg 197101, Russia; ilia.a.vovk@gmail.com (I.A.V.); lobano-vladimir@yandex.ru (V.V.L.); litvin88@gmail.com (A.P.L.); m.yu.leonov@gmail.com (M.Y.L.); a_v_fedorov@inbox.ru (A.V.F.); 2 The University of Sydney, Institute of Photonics and Optical Science (IPOS), School of Physics, Camperdown, NSW 2006, Australia

**Keywords:** quantum nanostructures, layered nanoplatelets, band structure, electron transition rates, intersubband optical transitions

## Abstract

This paper presents the first general theory of electronic band structure and intersubband transitions in three-layer semiconductor nanoplatelets. We find a dispersion relation and wave functions of the confined electrons and use them to analyze the band structure of core/shell nanoplatelets with equal thicknesses of the shell layers. It is shown that the energies of electrons localized inside the shell layers can be degenerate for certain electron wave vectors and certain core and shell thicknesses. We also show that the energies of intersubband transitions can be nonmonotonic functions of the core and shell thicknesses, exhibiting pronounced local minima and maxima which can be observed in the infrared absorption spectra. Our results will prove useful for the design of photonic devices based on multilayered semiconductor nanoplatelets operating at infrared frequencies.

## 1. Introduction

The confinement of charge carriers in semiconductor nanostructures endows them with physical properties that are not observed in the macroworld [1,2,3,4,5,6] and which can be controlled at the fabrication stage or by exposing fabricated nanostructures to external stimuli, such as electric [7,8,9,10,11,12] or magnetic [13,14,15,16] fields. The properties of semiconductor nanostructures can be altered in many ways, because they depend on the material parameters, size, and shape of the nanostructure. A celebrated example here is the emergence of giant optical activity in semiconductor nanocrystals with structural chirality [17,18,19,20,21,22,23,24,25]. The use of different semiconductors and the construction of heterojunctions significantly expand the scope of nanostructure design, allowing one, for example, to localize electrons and holes in certain parts of nanostructure to enhance the quantum yield of photoluminescence [26,27] or to increase the spatial separation of charge carriers for photovoltaic applications [28,29]. The development of nanotechnology has made possible the synthesis of nanoscale heterostructures of various geometries, including chiral quantum dots, layered nanoplatelets, tetrapods, and anisotropic nanorods [30,31,32]. Core/shell nanostructures, where one material (core) is isolated while another (shell) is exposed to the environment, are of particular practical importance [33,34,35].

Three-layer semiconductor nanoplatelets are one of the most practical and useful types of core/shell nanostructures [36,37,38]. Being quasi-two-dimensional structures, with quantum confinement perpendicular to their plane and two heterojunctions at the interfaces between the semiconductor layers, such nanoplatelets can be conveniently modeled as quantum wells with complex profiles. One of the key features of semiconductor quantum wells is the existence of subbands of dimensional quantization in the electron energy spectrum. This feature makes possible direct intraband (intersubband) transitions which play a crucial role in the infrared nanophotonics [39,40,41,42,43]. The interpretation of intersubband absorption spectra of three-layer semiconductor nanoplatelets requires knowing their band structure and intersubband transition rates, which are determined by the geometric and material parameters of the nanoplatelets.

Here we present a first general theoretical model of the conduction band of three-layer semiconductor nanoplatelets, which can describe nanoplatelets made of different semiconductor layers of different thicknesses. The model allows us to find the dispersion equation, energy spectrum, and wave functions of the confined electrons. We also obtain a closed-form expression for intersubband transition rates, which generalizes our previous result for bilayer nanoplatelets [44] and allows one to model intersubband absorption spectra of three-layer semiconductor nanoplatelets. The developed theory is then employed to analyze the energy spectrum of symmetric core/shell nanoplatelets, reveal degeneracies in the spectrum, and demonstrate nontrivial dependencies of the electron’s energies on the core and shell thicknesses. The results of our work will prove useful in the design of infrared detectors and emitters and will improve our understanding of the optics of layered semiconductor nanostructures.

## 2. Results

**Figure 1:**
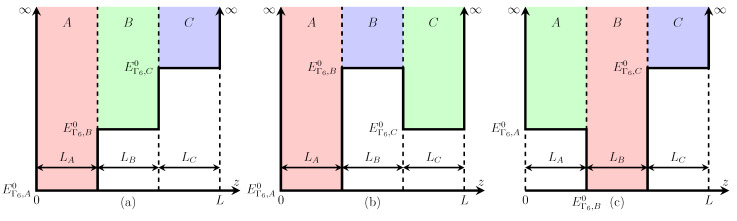
Three types of three-layer semiconductor nanoplatelets made of materials *A*, *B*, and *C* with different electron affinities $\chi_{A}$, $\chi_{B}$, and $\chi_{C}$: (**a**) $\chi_{A}>\chi_{B}>\chi_{C}$, (**b**) $\chi_{A}>\chi_{C}>\chi_{B}$, and (**c**) $\chi_{B}>\chi_{A}\;>\;\chi_{C}$.

Consider the conduction band of a three-layer nanoplatelet made of semiconductors *A*, *B*, and *C* with electron affinities $\chi_{A}$, $\chi_{B}$, and $\chi_{C}$. The nanoplatelet can be modelled as a two-dimensional quantum well with a complex profile and two heterojunctions at the interfaces between the layers. If the work functions of materials *A*, *B*, and *C* are alike, then the gaps between their conduction bands at the nanoplatelets interfaces are determined by the differences in the electron affinities of the adjacent materials. In the most general case of $\chi_{A}\neq \chi_{B}\neq \chi_{C}$, there are three types of nanoplatelets, as shown in Figure 1. We will analyze the structure of the conduction band in these nanoplatelets by assuming that the potential barriers for electrons at the nanoplatelet surface are infinite. This approximation is well justified for thick colloidal nanoplatelets made of narrow-bandgap semiconductors and suspended in a wide-bandgap dielectric medium, because the outer boundaries of such nanoplatelets are almost impenetrable for their confined electrons [45]. Its perfect validity for ultrathin single-layer colloidal nanoplatelets is demonstrated by an excellent agreement between the theoretical and experimental data in reference [46].

The band structures of many semiconductors can be conveniently described around the Γ point of the Brillouin zone using the eight-band Hamiltonian of the k·p perturbation theory [47]. For wide-bandgap semiconductors, we can neglect the influence of valence bands on the conduction band and use a simple two-band model of the $\Gamma_{6}$ conduction band (which is doubly degenerate over the spin). The spin-degenerate electron states have different Bloch amplitudes but the same envelope function $f_{\Gamma_{6}}\left(r\right)$ obeying the Schrödinger equation:

(1) $${\hat{H}}_{c}f_{\Gamma_{6}}\left(r\right)=E_{\Gamma_{6}}f_{\Gamma_{6}}\left(r\right),\;{\hat{H}}_{c}=\left\{\begin{array}{cc} E_{\Gamma_{6},A}^{0}+\hbar^{2}{\hat{k}}^{2}/\left(2m_{c,A}^{*}\right), & 0⩽z⩽L_{A},\\ E_{\Gamma_{6},B}^{0}+\hbar^{2}{\hat{k}}^{2}/\left(2m_{c,B}^{*}\right), & L_{A}⩽z⩽L_{A}+L_{B},\\ E_{\Gamma_{6},C}^{0}+\hbar^{2}{\hat{k}}^{2}/\left(2m_{c,C}^{*}\right), & L_{A}+L_{B}⩽z⩽L, \end{array}\right.$$

where $E_{\Gamma_{6}}$ is the energy of the electron in the conduction band, ${\hat{k}}^{2}$ is the square of the electron wave vector operator, and $L=L_{A}+L_{B}+L_{C}$ is the thickness of the nanoplatelet. This equation is equivalent to three particle-in-a-box problems in which the potential energies of the particle are given by the bottom energies $E_{\Gamma_{6},X}^{0}$ of the conduction band in the nanoplatelet’s layers, and the kinetic energies are characterized by the layer-dependent effective masses $m_{c,X}^{*}$(X=A,B,C).

In a situation wherein the lateral dimensions of the nanoplatelet are large compared to its thickness, the lateral confinement of electrons can be ignored and the envelope function can be written in the form

(2) $$f_{\Gamma_{6}}\left(r\right)=\frac{1}{\sqrt{S}}f_{\Gamma_{6}}\left(z\right)e^{i{\mathrm{kr}}_{\|}},$$

where *S* is the surface area of the nanoplatelet $\left(\sqrt{S}\gg L\right)$, $f_{\Gamma_{6}}\left(z\right)$ is the function of the transverse coordinate *z* only, $k=(k_{x},k_{y})$ is the in-plane wave vector of the electron, and $r_{\|}=\left(x,y\right)$ is the two-dimensional radius vector of the electron in the plane of the nanoplatelet.

Using Equation (1), one can show that the envelope function of the electron in layer *X* can be found from the equation

(3) $$\frac{d^{2}f_{\Gamma_{6},X}\left(z\right)}{dz^{2}}+\kappa_{X}^{2}f_{\Gamma_{6},X}\left(z\right)=0,$$

where

(4) $$\kappa_{X}^{2}=\frac{2m_{c,X}^{*}}{\hbar^{2}}\left(E_{\Gamma_{6}}-E_{\Gamma_{6},X}^{0}-\frac{\hbar^{2}k^{2}}{2m_{c,X}^{*}}\right).$$

It is seen that $\kappa_{X}$ can be either real or imaginary depending on whether the energy $E_{\Gamma_{6}}$ of the electron in the nanoplatelet layer *X* is larger or smaller than the energy $E_{\Gamma_{6},X}^{0}+\hbar^{2}k^{2}/\left(2m_{c,X}^{*}\right)$ of the electron in bulk semiconductor *X*.

The general solution of Equation (3) is of the form $f_{\Gamma_{6},X}\left(z\right)=X_{1}e^{i\kappa_{X}z}+X_{2}e^{-i\kappa_{X}z}$, where constants $X_{1}$ and $X_{2}$ are determined by the boundary conditions and the normalization condition

(5) $$\int_{0}^{L}{\left|f_{\Gamma_{6}}\left(z\right)\right|}^{2}dz=1.$$

Since we have assumed that the boundaries of the nanoplatelet are impenetrable for the confined electrons, the envelope function must vanish at these boundaries:

(6) $$f_{\Gamma_{6},A}\left(0\right)=f_{\Gamma_{6},C}\left(L\right)=0.$$

At the interfaces between the semiconductor layers, the following Bastard boundary conditions are used [48]:

(7) $$\begin{array}{c} f_{\Gamma_{6},A}\left(L_{A}\right)=f_{\Gamma_{6},B}\left(L_{A}\right),\;\frac{1}{m_{c,A}^{*}}\frac{df_{\Gamma_{6},A}\left(z\right)}{dz}{|}_{z=L_{A}}=\frac{1}{m_{c,B}^{*}}\frac{df_{\Gamma_{6},B}\left(z\right)}{dz}{|}_{z=L_{A}}, \end{array}$$

(8) $$\begin{array}{c} f_{\Gamma_{6},B}\left(L_{A}+L_{B}\right)=f_{\Gamma_{6},C}\left(L_{A}+L_{B}\right),\;\frac{1}{m_{c,B}^{*}}\frac{df_{\Gamma_{6},B}\left(z\right)}{dz}{|}_{z=L_{A}+L_{B}}=\frac{1}{m_{c,C}^{*}}\frac{df_{\Gamma_{6},C}\left(z\right)}{dz}{|}_{z=L_{A}+L_{B}}. \end{array}$$

Equations (6)–(8) lead to the following dispersion relation:

(9) $$G_{A}^{+}G_{B}^{+}G_{C}^{+}=G_{A}^{-}G_{B}^{-}G_{C}^{-},$$

where

(10) $$G_{X}^{\pm }=\frac{\kappa_{X}}{\kappa_{B}}\frac{m_{c,B}^{*}}{m_{c,X}^{*}}\cos\left(\kappa_{X}L_{X}\right)\pm i\sin\left(\kappa_{X}L_{X}\right).$$

In contrast to bulk semiconductors, wherein the states of conduction electrons are described by three real quantum numbers $k_{x}$, $k_{y}$, and $k_{z}$, in a two-dimensional nanoplatelet such states are characterized by two in-plane numbers $k_{x}$ and $k_{y}$ and by an integer quantum number n=1,2,3,…. This follows from Equation (9) whose solution for a given wave vector k gives a series of roots, each of which corresponds to a subband of the conduction band. Note that since the electron motion in the plane of the nanoplatelet is isotropic, it is described by the modulus of the two-dimensional wave vector k=|k|.

The envelope function of electron in the three layers of the nanoplatelet is given by

(11) $$\begin{array}{cc} f_{\Gamma_{6},A}\left(z\right) & =2iA_{1}\sin\left(\kappa_{A}z\right), \end{array}$$

(12) $$\begin{array}{cc} f_{\Gamma_{6},B}\left(z\right) & =A_{1}\left(G_{A}^{+}e^{i\kappa_{B}\left(z-L_{A}\right)}-G_{A}^{-}e^{-i\kappa_{B}\left(z-L_{A}\right)}\right), \end{array}$$

(13) $$\begin{array}{cc} f_{\Gamma_{6},C}\left(z\right) & =2igA_{1}\sin\left[\kappa_{C}\left(L-z\right)\right], \end{array}$$

where the normalization constant (calculated in the Appendix A) is

(14) $$\begin{array}{c} A_{1}=(2L_{A}\left[1-\mathrm{sinc}(2\kappa_{A}L_{A})\right]+2L_{C}g^{2}\left[1-\mathrm{sinc}(2\kappa_{C}L_{C})\right]\\ +L_{B}\left\{2G_{A}^{+}G_{A}^{-}-\left[{\left(G_{A}^{+}\right)}^{2}e^{i\kappa_{B}L_{B}}+{\left(G_{A}^{-}\right)}^{2}e^{-i\kappa_{B}L_{B}}\right]\mathrm{sinc}\left(\kappa_{B}L_{B}\right)\right\}{)}^{-1/2}, \end{array}$$

sincx=sinx/x, and

(15) $$g=\frac{G_{A}^{+}e^{i\kappa_{B}L_{B}}-G_{A}^{-}e^{-i\kappa_{B}L_{B}}}{2i\sin(\kappa_{C}L_{C})}.$$

Knowing the energies, $E_{\Gamma_{6},n}\left(k\right)$, and envelope functions, $f_{\Gamma_{6},X,n}\left(k,z\right)$, of the conduction electrons, one can calculate the probabilities of intersubband infrared transitions induced in the nanoplatelet by an external electromagnetic field. In the dipole approximation, the matrix element of transition i→f is given by [44]

(16) $$M_{fi}=\delta_{\alpha_{f}\alpha_{i}}\delta_{k_{f}k_{i}}M_{n_{f}n_{i}}^{\left(0\right)}\left(k_{i}\right),\;M_{n_{f}n_{i}}^{\left(0\right)}\left(k\right)=-i\frac{e\hbar }{mc}\left(A_{z}^{A}\Theta_{fi}^{A}+A_{z}^{B}\Theta_{fi}^{B}+A_{z}^{C}\Theta_{fi}^{C}\right),$$

where $j=\{\alpha_{j},k_{j},n_{j}\}$ is the set of quantum numbers of the electron in the *j*th state, $\alpha_{j}=\pm 1/2$ is the spin projection, $\delta_{ij}$ is the Kronecker delta, −e and *m* are the charge and mass of a free electron, $A_{z}^{X}$ is the *z*-component of the vector potential of the electromagnetic field in layer *X* (in the Coulomb gauge), and

(17) $$\Theta_{fi}^{X}=\int_{z\in X}d\zeta \;f_{\Gamma_{6},X,n_{f}}^{*}\left(k,\zeta \right)\;\frac{df_{\Gamma_{6},X,n_{i}}\left(k,z\right)}{dz}{|}_{z=\zeta }.$$

The rate of intersubband transitions per unit area of the nanoplatelet is given by the Fermi golden rule

(18) $$W=\frac{2}{\hbar }\sum_{n_{f},n_{i}}\int_{0}^{\infty }|M_{n_{f}n_{i}}^{\left(0\right)}\left(k\right){|}^{2}\left[F_{n_{i}}\left(k\right)-F_{n_{f}}\left(k\right)\right]\delta \left[E_{\Gamma_{6},n_{f}}\left(k\right)-E_{\Gamma_{6},n_{i}}\left(k\right)-\hbar \omega \right]kdk,$$

where $F_{n}\left(k\right)$ is the Fermi–Dirac distribution and δ(x) is the Dirac delta function.

## 3. Discussion

The developed theory describes the lowest conduction band of three-layer nanoplatelets made of cubic semiconductors and allows one to analyze nanoplatelets of asymmetric profiles shown in Figure 1. However, since core/shell nanoplatelets with symmetric profiles are more and more often used in practice [49,50], we shall consider them in more detail.

The outer layers of symmetric core/shell nanoplatelets are made of the same material and have the same thickness. The dispersion equation in this case is simplified to the form

(19) $$2\cot(\kappa_{\mathrm{core}}L_{\mathrm{core}})=-h+1/h,$$

where

(20) $$h=\frac{\kappa_{\mathrm{shell}}}{\kappa_{\mathrm{core}}}\frac{m_{c,\mathrm{core}}^{*}}{m_{c,\mathrm{shell}}^{*}}\cot\left(\kappa_{\mathrm{shell}}L_{\mathrm{shell}}\right)$$

and we have introduced new notations $L_{\mathrm{shell}}=L_{1}=L_{3}$ and $L_{\mathrm{core}}=L_{2}$.

Equation (19) breaks up into two equations:

(21) $$\cot\left(\frac{\kappa_{\mathrm{core}}L_{\mathrm{core}}}{2}\right)=\frac{1}{h},\;\cot\left(\frac{\kappa_{\mathrm{core}}L_{\mathrm{core}}}{2}\right)=-h.$$

The first of them gives odd solutions of the dispersion equation, described by symmetric wave functions, whereas the second corresponds to even solutions and antisymmetric wave functions. It can be shown that g=1 for odd solutions (with n=1,3,5,…) and g=−1 for even solutions (with n=2,4,6,…), and the normalization coefficient is given by

(22) $$A_{1}={\left(4L_{\mathrm{shell}}\left[1-\mathrm{sinc}(2\kappa_{\mathrm{shell}}L_{\mathrm{shell}})\right]+2L_{\mathrm{core}}G_{\mathrm{shell}}^{+}G_{\mathrm{shell}}^{-}\left[1-{\left(-1\right)}^{n}\mathrm{sinc}\left(\kappa_{\mathrm{core}}L_{\mathrm{core}}\right)\right]\right)}^{-1/2},$$

where, as before, *n* is the subband number and $G_{\mathrm{shell}}^{\pm }$ is defined by Equation (10). It should be noted that according to Equations (16) and (17), transitions between the subbands of the same parity are forbidden in symmetric nanoplatelets.

To illustrate the developed theory, we consider core/shell nanoplatelets made of three 7-nm-thick alternating layers of ZnSe and CdTe. The bottom of the conduction band in ZnSe (relative to the vacuum energy) is higher than that in CdTe; therefore, ZnSe represents a potential barrier for electrons inside the nanoplatelet. The gap between the conduction bands of ZnSe and CdTe is $\Delta E_{\Gamma_{6}}^{0}\;=\;0.22$ eV [32,51]. The effective masses of conduction electrons near the Γ point are 0.137 m for ZnSe and 0.09 m for CdTe [51].

**Figure 2:**
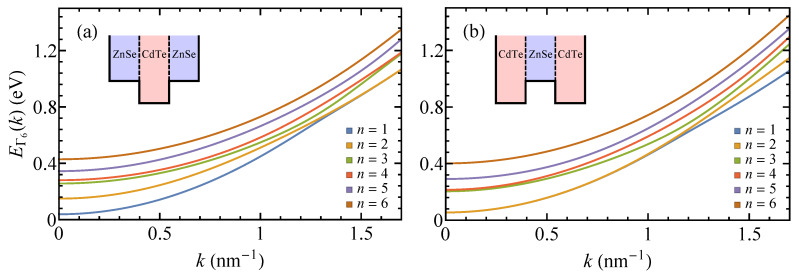
First six subbands of (**a**) ZnSe/CdTe/ZnSe and (**b**) CdTe/ZnSe/CdTe nanoplatelets with $L_{\mathrm{core}}=L_{\mathrm{shell}}=7$ nm. The energy is measured from the bottom of the conduction band of bulk CdTe. Note that the k·p perturbation theory gives the most accurate results in the vicinity of the Γ point, where the perturbation term is much smaller than the band gap energy (1.51 eV for CdTe) [53]. The material parameters for ZnSe and CdTe were taken from reference [51].

Figure 2 shows the subbands of ZnSe/CdTe/ZnSe and CdTe/ZnSe/CdTe nanoplatelets. The first two subbands of the ZnSe/CdTe/ZnSe nanoplatelet are seen to lie below the potential barrier $\Delta E_{\Gamma_{6}}^{0}$ near the Γ point (k=0), implying that the electrons with small *k*s are localized inside the core layer. Electrons can delocalize upon transition to higher subbands, as was shown in reference [44]. An interesting feature of the subbands of the nanoplatelet with a CdTe core is that they become degenerate in pairs for sufficiently large *k*s. A similar degeneration is observed in the vicinity of the Γ point for the low-energy bands of the nanoplatelet with a ZnSe core. This degeneration indicates that the electrons of the degenerate subbands are localized inside the shell of the nanoplatelet. The states of such electrons are described by wave functions which are symmetric and antisymmetric combinations of the wave functions of the two quantum wells provided by the shell layers and which only slightly penetrate into the core layer. The subbands of both nanoplatelets become degenerate when $E_{\Gamma_{6}}\left(k\right)⩽E_{\Gamma_{6},\mathrm{core}}^{0}+\hbar^{2}k^{2}/\left(2m_{c,\mathrm{core}}^{*}\right)$. Note that in contrast to core-only nanoplatelets, the intersubband transition energies of core/shell nanoplatelets depend on the electron wave vector, which can lead to the broadening of the transition spectra [52].

**Figure 3:**
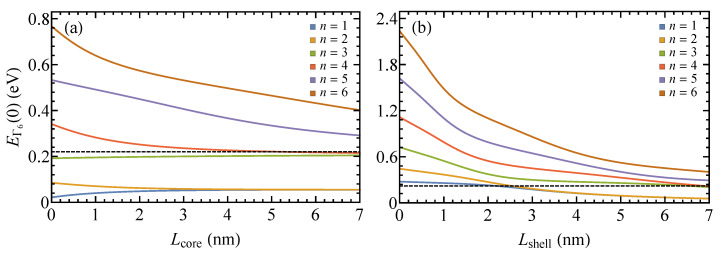
Energies $E_{\Gamma_{6}}\left(k=0\right)$ of the first six subbands of CdTe/ZnSe/CdTe nanoplatelets as functions of (**a**) core thickness $L_{\mathrm{core}}$ for $L_{\mathrm{shell}}=7$ nm and (**b**) shell layer thickness $L_{\mathrm{shell}}$ for $L_{\mathrm{core}}=7$ nm. The energy is measured from the bottom of the conduction band of bulk CdTe; dashed line is the barrier energy $\Delta E_{\Gamma_{6}}^{0}$. The material parameters are the same as in Figure 2.

Knowing how the band structure of layered nanoplatelets depends on their geometrical parameters is the key to designing photonic and optoelectronic devices based on intersubband transitions. The more complex the structure of the nanoplatelet, the more nontrivial this dependence can be. Let us consider the effect of the nanoplatelets’ geometry on the structure of their conduction band by the example of a CdTe/ZnSe/CdTe core/shell nanoplatelet. Figure 3 shows how the energies of its subbands depend on the thicknesses of its core and shell layers. When the shell thickness is zero (i.e., when the nanoplatelet is made of one semiconductor) the energies of the confined electrons obey the well-known relation $E_{\Gamma_{6},X}^{0}+{\left(\pi \hbar n\right)}^{2}/\left(2m_{c,X}^{*}L^{2}\right)$. Both kinds of dependencies shown in the figure are seen to noticeably deviate from this simple behavior $\left(\propto n^{2}/L^{2}\right)$. In particular, the energies of the first and third subbands in Figure 3a grow with the core thickness, and the energy of the third subband increases weakly and approximately linearly. The regions of linear growth also exist for other subbands; e.g., for the fifths subband when the shell thickness is between zero and 5 nm. Subbands 4, 5, and 6 in Figure 3b can be approximated by two linear functions: these functions have the same slope for $L_{\mathrm{shell}}$ from zero to 1–2 nm, whereas for larger $L_{\mathrm{shell}}$, their slope becomes smaller and does not change until the shell thickness reaches 4–5 nm.

As we have seen, the first and second subbands of the nanoplatelets with $L_{\mathrm{core}}=L_{\mathrm{shell}}>4$ nm are degenerate. This degeneracy is gradually lifted with the decrease in the core thickness for a fixed $L_{\mathrm{shell}}$ and with the decrease in the shell thickness for a fixed $L_{\mathrm{core}}$. In the first instance, the subbands become nondegenerate where their dispersion curves cross the barrier energy $\Delta E_{\Gamma_{6}}^{0}$. Equation (18) shows that the rate of intersubband transition 1→2 would be the largest in the core-only nanoplatelets $\left(L_{\mathrm{shell}}=0\right)$ at low temperatures. The strong dependence of the energy of this transition on the core and shell thicknesses allows one to adjust this energy as desired for applications. For example, as evidenced by Figure 3b, the transition energy reduces from 0.17 eV when $L_{\mathrm{shell}}=0$ to almost zero for $L_{\mathrm{shell}}>3$ nm when $L_{\mathrm{core}}=7$ nm.

Another interesting feature of the electron energy spectrum is the existence of local minima and maxima in the energies of some intersubband transitions as the functions of shell thickness. In particular, the energy of transition 2→3 has a local minimum of 0.1 eV at $L_{\mathrm{shell}}=2.25$ nm and a local maximum of 0.17 eV at $L_{\mathrm{shell}}=5.55$ nm. The absolute maximum of 0.28 eV is achieved at zero shell thickness. The local minima and maxima also exist for transitions 1→3 and 2→4, which, however, do not contribute to the interband absorption spectrum. A similar nonmonotonic dependence of transition rates on the shell thickness has been recently observed for Auger recombination in CdSe/CdS core/shell nanoplatelets [54].

As a concluding remark, we would like to discuss the applicability domain of our model and its ability to describe experimental data. The key simplifications used in this paper have been the two-band approximation of the electronic structure of semiconductor layers and the implicit neglect of the dielectric confinement and interface effects. The two-band approximation is widely used and suits well to describe the conduction band of nanoscale systems made from wide-bandgap II–VI semiconductors, such as CdSe, CdS, and CdTe. It gives accurate results even for semiconductor quantum dots, where the effect of size quantization is much stronger than in quantum wells and nanoplatelets [48,55,56,57]. However, if the nanoplatelets are made from narrow-bandgap semiconductors, such as InSb, InAs, PbS, PbSe, and PbTe, an eight-band model is needed to describe the conduction band.

Besides the quantum confinement due to the band discontinuities at the interfaces and boundaries of the nanoplatelet, the conduction electrons suffer the dielectric confinement due to the difference in the permittivities of the nanoplatelet and its surrounding. The dielectric confinement modifies the Coulomb interaction between the electrons and holes and changes the effective bandgap [58]. We could describe these effects as the interaction of electron with its image charge in the dielectric medium and incorporate them in the developed theory by adding a self-interaction term to the Hamiltonian. However, the resulting effect would likely to be negligible. First of all, because the decrease in the electron and hole energies due to the self-interaction is almost completely canceled when considering the interband transitions by the increase of the electron-hole binding energy [46]; but most importantly, because the dielectric confinement changes the absolute values of the electron energies, shifting all the dispersion curves by the same amount, and therefore, not affecting the intersubband transition energies or the shape of the optical spectrum.

Our second implicit assumption of the absence of trap states on the surface of the nanoplatelet is well justified in light of the very many strategies that have been developed to passivate the surface. The surface passivation is a key step of the nanoplatelet synthesis for optoelectronic applications because trap states can dramatically reduce the quantum yield of the nanoplatelets. This assumption is also supported by the fact that the electron wave functions can hardly penetrate the wide-bandgap medium, and the matrix elements of transitions to the trap states are negligibly small. In case of incompletely passivated nanoplatelets, a more sophisticated model is required to account for the effects of the remaining surface traps.

## 4. Conclusions

We have theoretically analyzed the electronic energy spectrum and intersubband transitions of three-layer semiconductor nanoplatelets. The dispersion equation and envelope wave functions of electrons confined by the nanoplatelets were derived analytically. By considering symmetric core/shell nanoplatelets made of alternating ZnSe and CdTe layers as an example, we showed that their subbands can be degenerate due to the strong localization of electrons inside the shell. This degeneracy was found to occur near the Brillouin zone center for the low-energy subbands if the affinity of electron in the core is less than that in the shell and at sufficiently large electron wave vectors for all subbands in the opposite case. It was also shown that the dependencies of the subband energies on the thicknesses of the core and shell layers may significantly differ from the similar dependence in a single-layer nanoplatelet. On the one hand, while in three-layer nanoplatelets the energies of some subbands can grow with core thickness, in single-layer nanoplatelets they always decrease. On the other hand, when considered as functions of shell thickness, the energies of subbands can change quasi-linearly and the energies of transitions between the subbands can have local minima and maxima. The results of this work can be used for modeling the intersubband spectra of symmetric and asymmetric three-layer quantum wells and for the development of novel devices for infrared nanophotonics.

## Abbreviations

$\chi_{X}$	electron affinity in layer *X*
$E_{\Gamma_{6}}$	energy of electron
$E_{\Gamma_{6},X}^{0}$	energy of the conduction band bottom in layer *X*
$m_{c,X}^{*}$	effective mass of electron in layer *X*
$L_{X}$	thickness of layer *X*
*L*	thickness of nanoplatelet
$f_{\Gamma_{6},X}$	envelope function of electron in layer *X*
*S*	surface area of nanoplatelet
r	radius vector of electron
$r_{\|}$	two-dimensional radius vector of electron in the plane of nanoplatelet
k	two-dimensional wave vector of electron
*k*	absolute value of two-dimensional wave vector of electron
$\kappa_{X}$	*z*-component of electron wave vector in layer *X*
$M_{fi}$	matrix element of electron transition i→f
*W*	intersubband transition rate per unit area of nanoplatelet

## Appendix A. Calculation of Normalization Constant

In order to derive Equation (14), one needs to calculate the normalization integral given in Equation (5). When evaluating this integral, we take into account that cosw, sinw, and $e^{iw}$ are *holomorphic* functions obeying the following equations:

(A1) $$\begin{array}{c} \cos^{*}w=\cos w^{*}=\cos\left(we^{-2i\phi }\right),\;\sin^{*}w=\sin w^{*}=\sin\left(we^{-2i\phi }\right), \end{array}$$

(A2) $$\begin{array}{c} \exp^{*}\left(iw\right)=\exp\left(-iw^{*}\right)=\exp\left(-iwe^{-2i\phi }\right), \end{array}$$

where $w=\left|w\right|e^{i\phi }$ is a complex number. Because $\kappa_{X}$ can be either real or imaginary, these equations yield:

(A3) $$\cos^{*}\left(\kappa_{X}z\right)=\cos\left(\kappa_{X}z\right),\;\sin^{*}\left(\kappa_{X}z\right)=\theta_{X}\sin\left(\kappa_{X}z\right),\;{\left(e^{i\kappa_{X}z}\right)}^{*}=e^{-i\theta_{X}\kappa_{X}z},$$

where $\theta_{X}=1$ for real $\kappa_{X}$ and $\theta_{X}=-1$ for imaginary $\kappa_{X}$. Using this result, we can transform the complex conjugate of the coefficient defined in Equation (10) as

(A4) $${\left(G_{X}^{\pm }\right)}^{*}=\frac{\theta_{X}\kappa_{X}}{\theta_{B}\kappa_{B}}\frac{m_{c,B}^{*}}{m_{c,X}^{*}}\cos\left(\kappa_{X}L_{X}\right)\mp i\theta_{X}\sin\left(\kappa_{X}L_{X}\right)=\theta_{X}\theta_{B}G_{X}^{\mp \theta_{B}},$$

where $G_{X}^{\mp \theta_{B}}=G_{X}^{\mp }$ for $\theta_{B}=1$ and $G_{X}^{\mp \theta_{B}}=G_{X}^{\pm }$ for $\theta_{B}=-1$.

The left hand side of Equation (5) splits into three integrals over the three layers of the nanoplatelet. Using Equations (A3) and (A4), the first integral can be calculated as

(A5) $$\begin{array}{c} |A_{1}{|}^{-2}\int_{0}^{L_{A}}|f_{\Gamma_{6},A}\left(z\right){|}^{2}dz=4\int_{0}^{L_{A}}{\left|\sin\left(\kappa_{A}z\right)\right|}^{2}dz\\ =4\theta_{A}\int_{0}^{L_{A}}\sin^{2}\left(\kappa_{A}z\right)\;dz=2\theta_{A}L_{A}\left[1-\mathrm{sinc}\left(2\kappa_{A}L_{A}\right)\right]. \end{array}$$

The second integral is given by

(A6) $$\begin{array}{c} |A_{1}{|}^{-2}\int_{0}^{L_{B}}|f_{\Gamma_{6},B}\left(z+L_{A}\right){|}^{2}dz=\int_{0}^{L_{B}}|G_{A}^{+}e^{i\kappa_{B}z}-G_{A}^{-}e^{-i\kappa_{B}z}{|}^{2}dz=-\theta_{A}\int_{0}^{L_{B}}{\left(G_{A}^{+}e^{i\kappa_{B}z}-G_{A}^{-}e^{-i\kappa_{B}z}\right)}^{2}dz\\ =\theta_{A}L_{B}\left\{2G_{A}^{+}G_{A}^{-}-\left[{\left(G_{A}^{+}\right)}^{2}e^{i\kappa_{B}L_{B}}+{\left(G_{A}^{-}\right)}^{2}e^{-i\kappa_{B}L_{B}}\right]\mathrm{sinc}\left(\kappa_{B}L_{B}\right)\right\}, \end{array}$$

where we have taken into account that

(A7) $${\left(G_{A}^{+}e^{i\kappa_{B}z}-G_{A}^{-}e^{-i\kappa_{B}z}\right)}^{*}=\theta_{A}\theta_{B}\left(G_{A}^{-\theta_{B}}e^{-i\theta_{B}\kappa_{B}z}-G_{A}^{+\theta_{B}}e^{i\theta_{B}\kappa_{B}z}\right)=-\theta_{A}\left(G_{A}^{+}e^{i\kappa_{B}z}-G_{A}^{-}e^{-i\kappa_{B}z}\right),$$

and the third integral is

(A8) $$|A_{1}{|}^{-2}\int_{0}^{L_{C}}|f_{\Gamma_{6},C}\left(z+L_{A}+L_{B}\right){|}^{2}dz=2\theta_{C}L_{C}{\left|g\right|}^{2}\left[1-\mathrm{sinc}\left(2\kappa_{C}L_{C}\right)\right]=2\theta_{A}L_{C}g^{2}\left[1-\mathrm{sinc}\left(2\kappa_{C}L_{C}\right)\right],$$

where we have used Equation (A7) with $z=L_{B}$ to get

(A9) $$g^{*}=\theta_{A}\frac{G_{A}^{+}e^{i\kappa_{B}L_{B}}-G_{A}^{-}e^{-i\kappa_{B}L_{B}}}{2i\theta_{C}\sin\left(\kappa_{C}L_{C}\right)}=\theta_{A}\theta_{C}g.$$

By summing up the three integrals, we finally get

(A10) $$\begin{array}{c} \theta_{A}{\left|A_{1}\right|}^{-2}=2L_{A}\left[1-\mathrm{sinc}(2\kappa_{A}L_{A})\right]+2L_{C}g^{2}\left[1-\mathrm{sinc}(2\kappa_{C}L_{C})\right]\\ +L_{B}\left\{2G_{A}^{+}G_{A}^{-}-\left[{\left(G_{A}^{+}\right)}^{2}e^{i\kappa_{B}L_{B}}+{\left(G_{A}^{-}\right)}^{2}e^{-i\kappa_{B}L_{B}}\right]\mathrm{sinc}\left(\kappa_{B}L_{B}\right)\right\}. \end{array}$$

Because the phase of $A_{1}$ does not affect measurable physical quantities, we omit $\theta_{A}$ here and arrive at Equation (14).
